# Regular Consumption of Lipigo^®^ Promotes the Reduction of Body Weight and Improves the Rebound Effect of Obese People Undergo a Comprehensive Weight Loss Program

**DOI:** 10.3390/nu12071960

**Published:** 2020-06-30

**Authors:** Marlhyn Valero-Pérez, Laura M. Bermejo, Bricia López-Plaza, Meritxell Aguiló García, Samara Palma-Milla, Carmen Gómez-Candela

**Affiliations:** 1 Nutrition Research Group, Hospital La Paz Institute for Health Research (IdiPAZ), 2804 Madrid, Spain; marlhyn.valero@idipaz.es (M.V.-P.); bricia.plaza@idipaz.es (B.L.-P.); 2 Nutrition Research Group, Hospital La Paz Institute for Health Research (IdiPAZ), Complutense University of Madrid, 28040 Madrid, Spain; 3 AB-BIOTICS SA, Av. Torre Blanca 57, 08172 Sant Cugat del Valles, Spain; aguilo@ab-biotics.com; 4 Nutrition Department, La Paz University Hospital, Nutrition Research Group, Hospital La Paz Institute for Health Research (IdiPAZ), Autonomous University of Madrid, 28046 Madrid, Spain; samara.palma@salud.madrid.org (S.P.-M.); cgcandela@salud.madrid.org (C.G.-C.)

**Keywords:** obesity, overweight, beta-glucans, chitosan, follow up study, weight loss programs, weight gain, weight loss, body weight changes

## Abstract

Obesity is a global public health problem. Objective: To evaluate the effect of the regular consumption of the product Lipigo^®^ on body weight and rebound effect on overweight/obese subjects undergoing a comprehensive weight loss program. Methods: A randomized, parallel, double-blind, placebo-controlled clinical trial was conducted with male and female subjects presenting a BMI 25–39.9 kg/m^2^. All subjects underwent a comprehensive weight loss program (WLP) for 12 weeks, which included an individualized hypocaloric diet, physical activity recommendations, nutritional education seminars, and three times a day consumption of the product Lipigo^®^ or Placebo. After-WLP, subjects continued the treatment for 9 months to assess rebound effect. Body weight (BW), BMI, and body composition were measured at the beginning and the end of the WLP, and in the follow-up. Results: A total of 120 subjects (85% women) 49.0 ± 9.5 years old and with a BW of 81.57 ± 13.26 kg (BMI 31.19 ± 3.44 kg/m^2^) were randomized and 73 subjects finished the study. At the end of the WLP, there was a tendency toward reduced BW (*p* = 0.093), BMI (*p* = 0.063), and WC (*p* = 0.059) in the treated group. However, subjects with obesity type 1 (OB1) from the treated group significantly reduced body weight (−5.27 ± 2.75 vs. −3.08 ± 1.73 kg; *p* = 0.017) and BMI (−1.99 ± 1.08 vs. −1.09 ± 0.55 kg/m^2^; *p* = 0.01) compared with placebo. They also presented a minor rebound effect after 9 months with product consumption (−4.19 ± 3.61 vs. −1.44 ± 2.51 kg; *p =* 0.026), minor BMI (−1.61 ± 1.43 vs. −0.52 ± 0.96 kg/m^2^; *p* = 0.025) and tended to have less fat-mass (−3.44 ± 2.46 vs. −1.44 ± 3.29 kg; *p* = 0.080) compared with placebo. Conclusions: The regular consumption of the product Lipigo^®^ promotes the reduction of body weight and reduces the rebound effect of obese people after 52 weeks (12 months), mainly in obesity type 1, who undergo a comprehensive weight loss program.

## 1. Introduction

Obesity is a major public health problem that has attained epidemic levels [1]. According to a recent estimate based on population analyses from 195 countries, 603.7 million adults, and 107.7 million children suffered obesity in 2015 worldwide [2]. The mean body mass index (BMI) is calculated to increase by 0.4 and 0.5 points in men and women, respectively, every decade [3]. Metabolic complications of obesity comprise metabolic processes dysfunction such as those controlling blood glucose, lipids, and pressure.

Severe dysregulation of these pathways give rise to a cluster of conditions known as metabolic syndrome [4,5]. As a result, around 3.4 million people die each year due to an overweight or obese condition [6].

Overweight and obesity are partially derived from a dietary energy imbalance stemming from behavioral, biological, and environmental processes [7]. Although not exclusively, obesity is tightly linked with hypercaloric diets, and thus weight reduction strategies are primarily focused on reducing energy consumption (i.e., dieting and reducing fat intake) and enhancing energy expenditure (i.e., regular exercise) [8]. Nevertheless, weight lowering methods based on diet and/or physical activity often fail to ameliorate obesity condition in a long-term period. Furthermore, most studies show weight regain upon medium to long-term follow-up (rebound effect) [9,10,11]. This phenomenon is likely multifactorial and can be explained by a poor compliance or by behavioral or physiological adaptations and highlights the importance of extending the follow-up period in interventional trials to adequately assess efficacy. 

Although a wide range of nutritional interventions pursuing weight loss are nowadays available in the market, evidence of their efficacy is fraught with uncertainty [12], and thus more adequately powered randomized trials with extended follow-up are required. Yeast-derived products as a source of biologically active oral compounds are recently gaining scientific support [13]. In vitro and in vivo data suggest that yeast-derived products, particularly of baker’s and brewer’s yeast *Saccharomyces cerevisiae*, harbor antioxidant, and free radical-scavenging properties besides the ability to stimulate the immune system [14,15]. Moreover, some clinical trials suggest that yeast hydrolysates may be a useful tool to help manage body weight and fat accumulation [16,17,18].

Lipigo^®^ is a polysaccharide-rich fraction containing β-glucan, chitin, and chitosan, obtained by a specific hydrolysis procedure of residual *S. cerevisiae* from brewery. A previous randomized, double-blind, placebo-controlled small trial demonstrated a statistically significant benefit of Lipigo^®^ on body weight management in overweight and obese population over 12 weeks [19,20]. In the present study, we sought to replicate the previous findings in a more robust randomized clinical trial, comprising a significantly bigger sample size, a better dietary control, and a longer treatment and follow-up periods. Moreover, we aimed to identify the population group able to obtain the greatest advantage of the oral intake of Lipigo^®^.

## 2. Materials and Methods

The present study was registered at http://clinicaltrials.gov under the number NCT03554525.

### 2.1. Study Subjects

Two hundred and twenty-four men and women aged 45–65 years were screened for the present study. Inclusion criteria were as follows: age between 18 and 65 years, BMI of ≥2 7 and <40 kg/m^2^, willing to follow a balanced hypocaloric diet to lose weight and perform regulated physical activity, absence of familial or social environment that prevents compliance with dietary treatment, having a suitable understanding of the clinical trial, agreeing to voluntarily participate in the study, and signing the informed consent form. The exclusion criteria were as follows: treatment for CV risk (dyslipidemia, hypertension, diabetes mellitus, and others), mental illness or low cognitive ability, history of severe liver or kidney disease or cancer, pregnancy or lactation, plans to stop smoking or to lose weight, allergy to any of the compounds of Lipigo^®^ as well as subjects who consumed >30 g/day alcohol, subjects were also excluded if they had participated in any program or clinical trials of weight control within the last 6 months.

The trial was approved by the Scientific Research and Ethics Committee of the HULP (Reference number: 4801) in accordance with the International Conference on Harmonization Guidelines on Good Clinical Practice and the ethical standards of the Declaration of Helsinki [21].

### 2.2. Study Design

This randomized, double-blinded, placebo-controlled clinical trial with two parallel arms was conducted at the Nutrition Department of La Paz University Hospital (HULP), Madrid (Spain). The total length of the intervention was 52 weeks (12 months). The intervention was divided in two phases: a weight-loss intervention phase (WLP) (12 weeks) in which all subjects were included in a dietary program controlled in 6 visits taking place every two weeks (V0–V6); and a follow-up post-weight lost intervention phase (P–WLP) (40 weeks) controlled in 3 visits taking place every three months (V7–V9). During both phases (WLP and P-WLP) participants were randomized with sex stratification to consume 3 sticks/day of Lipigo^®^ or Placebo (2 sticks just before the lunch and 1 stick just before the dinner).

### 2.3. Treatments

#### 2.3.1. Dietary Program

Hypocaloric diets (between 1500 and 3000 Kcal) were prescribed individually for all participants by a dietician expert at the Department of Nutrition of La Paz University Hospital, Madrid. Diets were designed to provide 30% less energy than the total energy expenditure (TEE) at baseline being 1500 kcal the lower limit for caloric restriction. Basal metabolic rate (BMR) was measured by bioelectric impedance Electro Fluid Graph + (EFG) (Akern s.r.l., Florence, Italy). BMR and TEE calculations were corrected according to physical activity and sex as recommended by the World Health Organization (WHO). Proposed hypocaloric diet consisted of 50–55% carbohydrates from total energy intake (added sugars <10%) and 29–34% fat (saturated fatty acids <10%, polyunsaturated fatty acids 5–10% and monounsaturated fatty acids, mainly from virgin olive oil, to complete the lipid profile), according to the recommendations of the Spanish Society of Community Nutrition (SENC, according to its Spanish initials, [22]). Proteins represented 20% of total energy intake (between 0.9–1.8 g/kg of body weight/day), based on body composition benefits observed in a recent meta-analysis [23]. The food intake was distributed in 5 meals: 3 main meals (breakfast, lunch, and dinner) and 2 snacks (mid-morning (11:00 a.m.–11:29 a.m.), and afternoon (5:00–5:29 p.m.)).

The hypocaloric dietary program was prescribed at baseline (V0) of the WLP: participants received a 7-day-meal plan as an example of the individualized diet designed for each one. Moreover, each participant received a food exchange list, allowing the personalization of diet plans according to individual preferences, but ensuring that the resulting menu would provide the individual nutritional requirements calculated. Further dietary counseling was given every two weeks (V1-V5) until the end of the WLP (12th week, V6). Dieticians used all these visits to resolve questions and to motivate participants sufficiently to comply with dietary advice. All subjects were given recommended portion sizes and information on possible food swaps. Moreover, nutrition education and motivational sessions were given by the dietician.

#### 2.3.2. Physical Activity Recommendations

In V0 subjects were instructed to perform moderate physical activity for 1 h at least 3 times a week. The subjects began according to their level of physical activity and gradually increased until they achieved 3 sessions per week or more at the end of WLP.

#### 2.3.3. Nutrition and Health Education Sessions

During the WLP, participants attended 5 nutrition and health education sessions (visits 1 to 5) goals to promote healthy eating and physical activity.

#### 2.3.4. Lipigo^®^ or Placebo

During the WLP and P-WLP, subjects consumed 3 sticks/day of Lipigo^®^ or Placebo (2 sticks just before the lunch and 1 stick just before the dinner)

Lipigo^®^ is a fiber combination obtained from *S. cerevisiae* from the brewery industry. Each stick contained a polysaccharide-rich fraction (909 mg β-glucan, 91 mg chitin-chitosan) and 400 mg excipients. The polysaccharide fraction was obtained by a specific hydrolysis procedure of residual *S. cerevisiae* from brewery patented by DAMM S.A (El Prat del Llobregat, Barcelona, Spain). The nutritional composition per 100 g of dry product was: protein, 1.6 g; fat, 3.7 g; carbohydrates 58.7 g; dietary fiber, 29.9 g; and sodium 0.6 g.

Placebo was composed of 1000 mg maltodextrine and 400 mg excipients.

DAMM S.A. prepared the Lipigo^®^ and the Placebo sticks specifically for this study. Both types of sticks were packaged in box packs of 30 sticks. The packs were labeled as either L1 or L2 to maintain blinded conditions. During every visit in the WLP, subjects received all the sticks needed until the next visit every two weeks. At baseline of the P-WLP (V6) and in the V7 and V8, subjects received all the sticks need to complete three months to the next visit. The sticks received by each participant were assigned according to the randomization.

### 2.4. Endpoints

The following analyses and measurements were collected:

#### 2.4.1. General Health Status Variables

During both phases (V0–V9), information about special medical conditions and drug consumption was collected. Furthermore, blood pressure and heart rate were measured using a Spot Vital Signs 420 automatic monitor (Welch Allyn, Madrid, Spain; accuracy ±5 mmHg). Three measurements were taken at 5-min intervals, and the means were calculated. Moreover, blood sample was collected to analyze lipid profile including total cholesterol and LDL-cholesterol at the beginning and at the end of each phase (V0, V6, and V9).

#### 2.4.2. Anthropometrics and Body Composition Variables

Anthropometric measurements were performed at the beginning and at the end of each phase (V0, V6, and V9) using standard techniques, adhering to WHO guidelines [24]. All measurements were made by trained personnel in the morning with the subject barefoot and wearing only underwear. Height was determined using a height meter with an accuracy of 1 mm (range, 80–200 cm). Body weight was measured using a TANITA BC-420 MA (Bio Lógica Tecnología Médica S.L, Barcelona, Spain). The BMI was calculated as body weight ((kg)/(height (m)^2^). According to their BMI, participants were classified in four categories: normal weight (BMI 18.5–24.9 kg/m ^2^), class I overweight (BMI 25.0–27.9 kg/m ^2^), class II overweight (BMI 28–29.9 kg/m ^2^), class I obesity (BMI 30–34.9 kg/m^2^), and class II obesity (BMI 35–39.9 kg/m^2^). Waist circumference (WC) was measured using a Seca 201 steel tape (Quirumed, Valencia, Spain). Variables change between V0 and V6 (Dif V0–V6) and between V6 and V9 (Dif V6–V9) was calculated in order to evaluate the anthropometrics and body composition evolution during WLP and P–WLP respectively. Variables change between V0 and V9 (Dif V0–V9) was calculated to evaluate the rebound effect.

Body composition was determined using a specialized tetrapolar bioelectrical impedance analyzer, the EFG ElectroFluidGraph analyzer (Akern s.r.l., Florence, Italy): total fat mass (TFM), fat-free mass (FFM), and muscle mass (MM) were measured. These body composition data were obtained using regression validated equations of the manufacturer (Akern BodyGram Plus 1.0). These validated equations were derived from previous research [25].

All participants were instructed by the researcher to minimize the BIA affecting factors (not having used diuretic medications in the previous seven days, to have been fasting for at least four hours; not having ingested alcohol or caffeinated beverages in the previous 48 h; having abstained from moderate-intense physical activity in the previous 24 h) [26].

The device was calibrated before measurements of each participant. All participants rested (lying on a bed) for at least five minutes prior to the measurement. Electrodes were placed on the dorsal surface of the wrist and the ankle as well as at the base of the second or third metacarpal-phalangeal joints of hand and foot after the skin was cleaned with an alcohol wipe. The lead wires were attached to the appropriate electrodes and participants were instructed to abduct their limbs from the trunk. Triplicate measurements were conducted, and a median value determined.

#### 2.4.3. Dietary Variables

The diet of each subject was recorded during WLP and P-WLP. In V0, V6–V9 participants filled a 24-h recall over 3 consecutive days recording all food and beverages consumed inside and outside the home, including one weekend day [27]. In V2-V5 diet was recorded using a 24 h record questionnaire. Food weight or household measurements (spoonful, cups, etc.) should be self-reported in both food recording questionnaires. Subjects were previously trained by the researchers to obtain accurate data. All food records were thoroughly reviewed by a nutritionist in the presence of the subject during study visits to ensure that the information collected was complete. The energy and nutritional content of the foods consumed were then calculated using DIAL software (Alce Ingeniería, Madrid, Spain).

#### 2.4.4. Physical Activity Variables

Physical activity was assessed using the International Physical Activity Questionnaire-Short Form (IPAQ-SF) for the Spanish population. IPAQ-SF collects the frequency and duration of vigorous-intensity activity, moderate-intensity activity, and walking activity [28]. The questionnaire was filled in V0, V6–V9. Time spent in vigorous, moderate, and walking activity was calculated by the energy spent for these categories of activity, to produce the total Metabolic Equivalent Task (MET)-minutes of physical activity/week.

#### 2.4.5. Compliance and Adverse Events

Subjects received the exact number of sticks required for each period during study visits. Empty and non-empty sticks should be returned to the investigator in each study visit. Compliance was measured by comparing the number of sticks provided and the number of empty sticks returned. A subject was considered compliant when he/she consumed ≥80% of the sticks provided. Adverse events were documented in all study visits (V1-V9). An adverse event was defined as any unfavorable, unintended effect. All such events were recorded along with the symptoms involved (bad breath, nausea, vomiting, diarrhea, constipation, others).

### 2.5. Statistical Analysis

The sample size was calculated taking into account the results obtained in a meta-analysis aiming to evaluate the effect of different non-surgical treatments on body weight maintenance in overweight/obese people [29]. Most of the studies included in such meta-analysis have a sample size of around 80 subjects. Moreover, according to these studies, a potential 33% drop-out range should be considered. Given all the above, the total sample size estimated for the present study was of 120 subjects.

The analysis population included all subjects who completed the WLP and P–WLP stages. Data is presented as mean ±standard deviation (SD) or percent (%) and N. The Kolmogorov-Smirnov test was used to check the normal distribution of the data. Outliers (i.e., lying more than two SDs from the mean) in asymmetric distributions were deemed to reflect true results and were included in the analysis. The Levene’s test was used to determine whether the variance presented by the measured variables was homogeneous. The Student t test was used to compare the mean values of normally distributed variables for the two treatment groups and the intragroup analysis. Whereas, Mann–Whitney U test was used when data were not normally distributed. Differences within groups between V0–V6, V6–V9 and V0–V9 were examined using the Student paired t test when the distribution of the results was normal and the Wilcoxon test when it was not. Additionally, Bonferroni’s correction for multiple comparisons was performed. All tests were two-tailed.

A subgroup of analysis was also conducted based on the presence of overweight or obesity at the baseline (V0). Significance was set at two-sided *p* < 0.05. All calculations were performed using SPSS v.21.0 software (SPSS Inc.).

## 3. Results

### 3.1. Recruitment and Study Population

**Figure 1:**
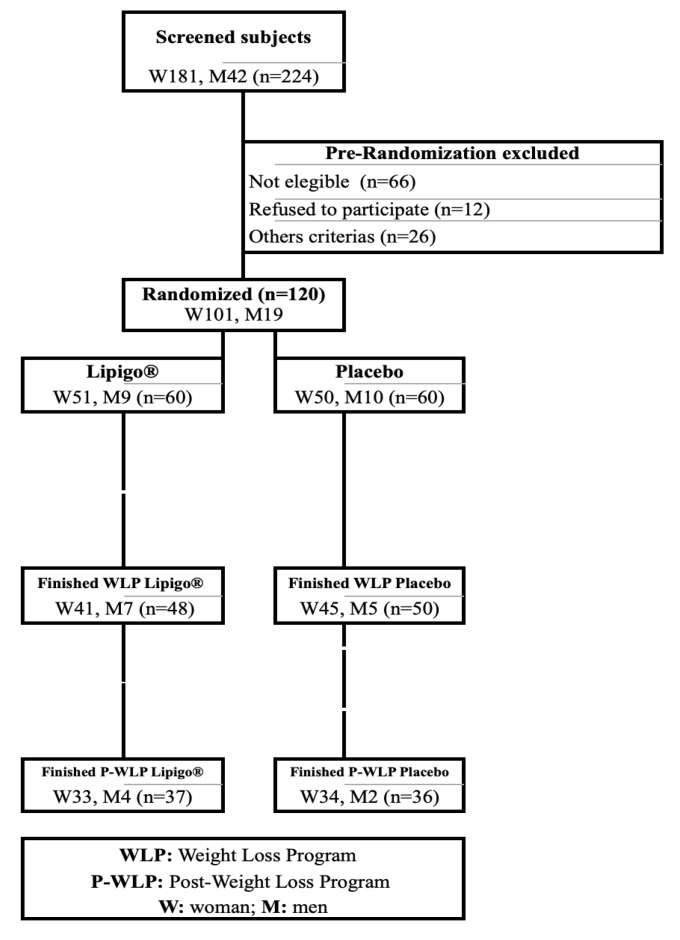
CONSORT diagram.

The study was performed between April 2017 and August 2018. One hundred and twenty apparently healthy subjects (19 men [15.8%], 101 women [84.2%]) were eligible for their inclusion in the study. Subjects were randomized into either the Placebo or Lipigo^®^ group stratified by sex. Twenty-two subjects dropped-out during the WLP:10 in the Placebo group and 12 in the Lipigo^®^ group. At the end of the study, a total of 47 subjects were lost to follow-up (24 in the Placebo group and 23 in the Lipigo^®^ group) due to personal causes (*n* = 27), failure to follow treatment instructions (*n* = 4), health problems not related to clinical trial procedures (*n* = 6), loss of follow-up (*n* = 9), and low product tolerance (*n* = 1). Thus, 73 subjects (6 men [8.2%], 67 women [91.8%]) completed the 12-months study, and only their results were included in the subsequent analyses (Figure 1).

### 3.2. Baseline Characteristics

The mean age of the population was 50.9 ± 8.9 years old. The mean BMI was 31.2 ± 3.5 kg/m^2^ (Obesity type 1, 39.7%). At baseline, no significant differences existed between subjects assigned to the Lipigo^®^ and Placebo groups in terms of their anthropometric, body composition, blood pressure, and biochemical parameters or other variables such as sex, age, or smoking (Table 1).

Despite existing differences between the number of women and men participating in the study, stratified randomization by sex allowed to obtain 2 homogeneous intervention groups.

### 3.3. Anthropometric and Body Composition Variables

Both treatment groups decreased body weight, BMI, and waist circumference at the end of WLP or P-WLP although no significant intra-group differences were detected in any anthropometric or body composition variables. No significant intergroup differences were obtained (Lipigo^®^ vs. Placebo, Table 2).

However, when evaluating the changes in anthropometric and body composition variables at the end of WLP (Dif. V0–V6), subjects included in the Lipigo^®^ group showed a non-significant trend towards reducing body weight (*p* = 0.093), BMI (*p* = 0.063), and waist circumference (*p* = 0.059) when compared to Placebo (Table 3).

A significant reduction of the lean mass was observed in subjects included in the Lipigo^®^ group when compared to Placebo during the P-WLP phase (Dif V6–V9, Lipigo^®^: −0.59 ± 1.57 vs. Placebo: 0.25 ± 1.52 kg; *p* = 0.024).

Next, subgroup analyses based on the presence of overweight type 2 (*n* = 32, 43.8%) or obesity type 1 (*n* = 29, 39.7%) and obesity type 2 (*n* = 12, 16.5%) was performed. Subjects with obesity type 1 and 2 enrolled in the Lipigo^®^ group showed a non-significant higher body weight reduction compared to Placebo at the end of WLP (Dif V0–V6, −5.24 ± 2.53 vs. −3.81 ± 2.2 kg *p* = 0.065). Nevertheless, when analyzing subjects with obesity type 1, Lipigo^®^ group, achieved a significantly higher body weight (−5.27 ± 2.75 vs. −3.08 ± 1.73 kg; *p* = 0.017) and BMI (−1.99 ± 1.08 vs. −1.09 ± 0.55 kg/m^2^; *p* = 0.010) reduction vs. Placebo at the end of WLP (Dif V0–V6). In addition, Lipigo^®^ group seemed to have a higher fat mass reduction than Placebo group at the end of WLP (Dif V0–V6) (−3.44 ± 2.46 vs. −1.44 ± 3.29 kg; *p* = 0.080). Moreover, intragroup difference V0 vs. V6 was observed in BMI only in Lipigo^®^ group (Table 4).

During the P-WLP (Dif v6–v9) both groups gain weight, BMI, and fat mass but the OB1 subjects included in the Placebo group gain more in all the anthropometric and body composition variables during this period (40 weeks) than Lipigo^®^ group, although the differences were not significant.

Moreover, obesity type 1 subjects included in the Lipigo^®^ group presented a significant minor rebound effect (Dif V0–V9) vs. Placebo group when looking at body weight change (−4.19 ± 3.61 vs. −1.44 ± 2.51 kg; *p* = 0.026) and BMI change (−1.61 ± 1.43 vs. −0.52 ± 0.96 kg/m^2^; *p* = 0.025). Lipigo^®^ group seemed to have a minor rebound effect (Dif V0–V9) in fat mass when compared to Placebo group (−1.62 ± 3.45 vs. 0.62 ± 3.80 kg) although the difference is no significant (*p* = 0.121).

### 3.4. Dietary Variables

At baseline, a mean general caloric intake of 1988.24 ± 437.13 kcal was documented. At the end of WLP both groups significantly reduced energy intake (Dif V0–V6) with no significant (NS) differences intergroup (−278.97 ± 460.7 vs. −288.82 ± 497.34 kcal; NS).

The caloric profile changed in both groups at the end of the WLP (Dif V0–V6) with no significant differences respected to baseline between Lipigo^®^ and Placebo groups: carbohydrates (0.64 ± 8.08 vs. 2.6± 5.98%; NS), proteins (1.56 ± 3.62 vs. 0.95 ± 2.83%; NS) and lipids (−1.78 ± 7.46 vs. −2.43 ± 5.24%; NS).

At the end of the P-WLP, no significant differences (Dif V6–V9) were observed in the caloric profile between Lipigo^®^ and Placebo groups when compared to baseline: carbohydrates (2.36 ± 7.89 vs. −0.63 ± 6.73%; NS), proteins (−1.64 ± 3.62 vs. −0.36 ± 3.16%; NS), and lipids (−0.29 ± 6.88 vs. 1.01 ± 6.41%; NS).

### 3.5. Physical Activity Variables

Regarding physical activity (total METs), both Lipigo^®^ and Placebo groups showed an increase in the WLP (Dif V0–V6, 530.37 ± 90.47 vs. 372.17 ± 981.91 METs; NS) and a decrease in P-WLP (Dif V6–V9, −186.48 ± 1035.93 vs. −224.89 ± 888.28 METS; NS). No significant differences were found between groups.

Specifically, sitting time was reduced in both Lipigo^®^ and Placebo groups at the end of the WLP (Dif V0–V6, −1.05 ± 2.53 vs. −0.56 ± 2.25 h; NS). On the other hand, walking time was as increased in both groups (12.62 ± 45.05 vs. 21.71 ± 55.13 min; NS). No significant differences were found between groups.

### 3.6. Compliance and Adverse Events

When analyzing compliance, 88.2 ± 8.04% of the participants showed proper adherence to treatment. No serious adverse events were observed with Lipigo^®^ consumption.

Bad breath, diarrhoea, constipation, bloating, nausea, and heartburn were documented as adverse events throughout the study. No differences were observed between treatment groups during all the study (V0–V9), except for Bloating having a significantly higher incidence in Lipigo^®^ vs. Placebo group in V2 only (24.3 vs. 2.9%; *p* = 0.008).

## 4. Discussion

This is the first randomized clinical trial conducted during 52 weeks (12 months) to study the effect of fibers combination supplement obtained from *S. cerevisiae* (Lipigo^®^) on weight loss after a 12 weeks Weight Loss Program (based in hypocaloric diet, physical activity recommendations, and nutritional education) and on rebound effect during 40 weeks follow-up.

The study demonstrates that three sticks/day of a polysaccharide-rich fraction (909 mg β-glucan, 91 mg chitin-chitosan) decrease significantly body weight and BMI after WLP only in OB1 subjects. Other BMI classifications (overweight or obese type II) reduce body weight and BMI classification but not significantly. Moreover, body weight, BMI, and waist circumference showed a slight reduction in Lipigo^®^ group compared to Placebo after WLP, independently of BMI classification.

In a previous double-blinded, randomized study, Santas et al. showed that Lipigo^®^ was beneficial in OB1 and overweight subjects. In that study, body weight and waist circumference were also reduced when compared to Placebo after 3 months of treatment. Importantly, participants were not enrolled in a body weight loss program [19]. Then, the present study results on body weight and waist circumference reduction support the efficacy of Lipigo^®^ even when combined with a controlled diet.

Furthermore, a possible role of fiber combinations including β-glucan and chitin-chitosan on body weight loss in obese and overweight populations has been widely studied by others. Pittler et al., was the first group to perform a randomized, double-blinded, Placebo-controlled trial providing data on BMI and body weight from overweight subjects after fiber intake [30]. However, their results showed a non-significant effect for 1g/day of chitin-chitosan fibers on body weight or BMI reduction after 4 weeks of treatment. Despite that, other groups have evidenced short-and mid-term effects on body weight and BMI loss after longer administration periods of fibers combination including chitin-chitosan [31]. Two independent groups have published significant body weight and BMI reduction in obese population concomitantly following a calorie restriction and physical activity program [32,33]. The study design followed on both studies is the most similar to the present study. Nevertheless, subjects enrolled in previous studies underwent longer dietary and physical activity programs when compared to the present study (12 or 6 months vs 3 months).

The present study also showed a lean mass decrease after WLP. This result could be associated with a good adherence to the WLP and Lipigo^®^ intake, instead of physical activity recommendations. Significant body weight loss was also achieved in other studies which provided a controlled diet but not physical activity advice [34,35,36]. However, the treatment period in these studies ranged from 2 to 6 months. In addition, the daily dosage of chitosan fibers (3 g) was supplemented with β-glucan and oat fibers more than betaine hydrochloride and aloe saponins in the Kaats et al. study [36]. Moreover, other studies have shown positive effects of a chitosan compound on the depletion of excess body fat under free-living conditions with minimal loss of fat-free or lean body mass [37,38]. Together, these findings suggest that β-glucan and/or chitosan combination fibers can impact body weight independently of physical activity.

To our knowledge, no previous studies have assessed the effect of β-glucan and chitosan treatments over rebound effect during a follow-up period after a weight loss program. The most highlighted result of this study is that OB1 subjects get the most benefit from consuming the product, not only during a 12 weeks weight loss program, but also decreasing the rebound effect especially in weight and BMI after 52 weeks (12 months) of starting the study including a 9 months follow-up period. This result could be due to the OB1 subjects included in the Lipigo group getting lower gain in anthropometric and body composition variables than the placebo groups during the 9 months follow-up period (P-WLP), although the differences were not significant.

OB1 subjects being the most benefited population from Lipigo^®^ treatment may have several explanations. On the one hand, overweight non-obese participants have been previously associated with less interest in body weight reduction than the obese population [39,40]. This fact may lead to decreased motivation to strictly follow a calorie restriction diet. On the other hand, greater amounts of fat may increase the difficulty of losing weight [41]. This particularity might be the reason OB2 subjects are not getting a benefit from product consumption. However, the small OB2 sample size does not allow any firm conclusions to be drawn in this subgroup.

One limitation of the present study was that body composition was measured by bioimpedance electrical. This method is not a gold standard but is a method relatively simple, inexpensive, and non-invasive technique suitable in field studies. In fact, the European Society of Parenteral and Enteral Nutrition (ESPEN) suggests that BIA works well to evaluate body composition in healthy and ill subjects (including overweight and obese people), using validated BIA equation appropriate to age, sex and race [42]. This aspect has been considered in the present study.

In summary, these results support the efficacy of Lipigo^®^ on body weight reduction as a concomitant and follow-up treatment to a controlled diet. Furthermore, we could for the first time demonstrate an important rebound effect reduction after 52 weeks (12 months) follow up period. Future studies with Lipigo^®^ aim to investigate the ability of the product to improve serum lipid profile and other biochemical and cardiovascular risk factors.

## 5. Conclusions

Regular consumption of Lipigo^®^ presented an adjuvant effect on body weight loss in the context of an individualized intervention, with a hypocaloric diet, physical activity practice, and nutritional education sessions. In this sense, the action of Lipigo^®^ is more evident in OB1 participants undergoing a weight control program. Moreover, in OB1 subjects, Lipigo^®^ intake reduced the rebound effect on body weight and BMI after 52 weeks (12 months) of starting the study including a 9 months follow-up period.

Based on the results obtained, the use of fiber mix from *S. cerevisiae* could be a complementary product to be included in weight control programs as well as in follow-up phases in obesity subjects.

## Figures and Tables

**Table 1:** Baseline characteristics of the subjects.

		Total	Placebo (*n* = 36)	Lipigo^®^ (*n* = 37)	*p*-Value
Gender	(Female %, n)	73	94.4 (34)	89.2 (33)	0.414
Age	(years)	50.9 ± 9.07	49.47 ± 10.4	52.3 ± 7.4	0.185
Weight	(kg)	82.85 ± 12.37	82.65 ± 12.5	83.04 ± 12.4	0.895
BMI	(kg/m^2^)	31.19 ± 3.44	31.2 ± 3.6	31.2 ± 3.3	0.976
Waist circumference	(cm)	97.76 ± 11.25	97.4 ± 11.6	98.2 ± 11.0	0.765
FM	(kg)	31.51 ± 7.65	32.3 ± 7.9	30.7 ± 7.5	0.400
Lean mass	(kg)	51.25 ± 10.15	50.3 ± 9.1	52.2 ± 11.1	0.439
MM	(kg)	35.21 ± 8.03	34.3 ± 6.8	36.2 ± 9.1	0.311
SBP	(mmHg)	111.47 ± 15.13	110.7 ± 15	112.3 ± 15.5	0.666
DBP	(mmHg)	77.19 ± 9.56	77.0 ± 9.7	77.4 ± 9.6	0.864

Data is represented as mean ± SD. BMI: Body Mass Index; FM: Fat Mass; MM: Muscle Mass; SBP: Systolic Blood Pressure; DBP: Diastolic Blood Pressure.

**Table 2:** Anthropometric and body composition variables throughout the study.

		Placebo (*n* = 36)	Lipigo^®^ (*n* = 37)	*p*-Value
Weight (kg)	Baseline of WLP (V0)	82.65 ± 12.48	82.65 ± 12.48	0.895
	End of WLP (V6)	79.19 ± 12.3	78.61 ± 12.56	0.842
	End of P-WLP (V9)	80.18 ± 12.49	79.67 ± 13.01	0.864
BMI (kg/m^2^)	Baseline of WLP (V0)	31.21 ± 3.65	31.18 ± 3.28	0.976
	End of WLP (V6)	29.91 ± 3.54	29.39 ± 3.19	0.512
	End of P-WLP(V9)	30.27 ± 3.62	29.79 ± 3.32	0.558
Waist circumference (cm)	Baseline of WLP (V0)	97.35 ± 11.6	98.18 ± 11.05	0.765
	End of WLP (V6)	94.82 ± 11.82	93.41 ± 10.36	0.588
	End of P-WLP (V9)	91.4 ± 17.54	93.94 ± 10.66	0.456
Muscle mass (kg)	Baseline of WLP (V0)	34.25 ± 6.84	36.19 ± 9.07	0.311
	End of WLP (V6)	32.12 ± 6.55	33.23 ± 8.42	0.530
	End of P-WLP (V9)	32.51 ± 6.84	32.62 ± 6.87	0.944
Lean mass (kg)	Baseline of WLP (V0)	50.32 ± 9.12	52.19 ± 11.14	0.439
	End of WLP (V6)	48.75 ± 8.65	49.61 ± 9.01	0.680
	End of P-WLP (V9)	49.00 ± 9.11	48.92 ± 9.07	0.971
Fat mass (kg)	Baseline of WLP (V0)	32.28 ± 7.86	30.74 ± 7.48	0.400
	End of WLP (V6)	30.47 ± 7.21	29.01 ± 6.41	0.365
	End of P-WLP (V9)	31.32 ± 7.34	30.78 ± 7.17	0.755

Data is expressed as mean ± SD.V: Visit; WLP: Weight Loss Program; P-WLP: Post-weight Loss Program; OB1: Obesity Type 1; BMI: Body Mass Index.

**Table 3:** Changes in anthropometric and body composition variables throughout the study.

		Placebo (*n* = 36)	Lipigo^®^ (*n* = 37)	*p*-Value
Weight (kg)	Dif V0–V6	−3.46 ± 2.00	−4.43 ± 2.78	0.093
	Dif V6–V9	0.99 ± 2.35	1.06 ± 2.87	0.912
Rebound effect	Dif V0–V9	−2.47 ± 3.41	−3.36 ± 3.36	0.259
BMI (kg/m^2^)	Dif V0–V6	−1.31 ± 0.77	−1.79 ± 1.35	0.063
	Dif V6–V9	0.36 ± 0.89	0.41 ± 1.09	0.831
Rebound effect	Dif V0–V9	−0.94 ± 1.32	−1.4 ± 1.58	0.178
Waist circumference (cm)	Dif V0–V6	−2.63 ± 3.1	−4.34 ± 4.13	0.059
	Dif V6–V9	−0.59 ± 3.3	0.53 ± 3.4	0.162
Rebound effect	Dif V0–V9	−3.08 ± 3.44	−3.48 ± 3.25	0.631
Muscle mass (kg)	Dif V0–V6	−2.14 ± 1.96	−2.04 ± 1.85	0.837
	Dif V6–V9	0.39 ± 1.57	−0.04 ± 1.36	0.218
Rebound effect	Dif V0–V9	−1.74 ± 2.23	−2.12 ± 1.74	0.444
Lean mass (kg)	Dif V0–V6	−1.57 ± 1.85	−1.5 ± 1.76	0.872
	Dif V6–V9	0.25 ± 1.52	−0.59 ± 1.57	0.024
Rebound effect	Dif V0–V9	−1.32 ± 1.99	−2.06 ± 1.84	0.108
Fat mass (kg)	Dif V0–V6	−1.81 ± 3.53	−2.6 ± 2.56	0.282
	Dif V6–V9	0.85 ± 2.5	1.59 ± 2.56	0.214
Rebound effect	Dif V0–V9	−0.96 ± 4.34	−1.06 ± 3.32	0.908

Data is expressed as mean ± SD. Dif V0–V6: Difference during the WLP; Dif V6–V9: Differences during the P-WLP; Dif V0–V9: Rebound effect. WLP: Weight Loss Program; P-WLP: Post-weight Loss Program; BMI: Body Mass Index.

**Table 4:** Changes in body weight, BMI, and Fat mass throughout the study in overweight (*n* = 32) and obesity type 1 (*n* = 29) subjects.

		Placebo	Lipigo^®^	*p*-Value
Weight (kg) **Overweight**	Baseline of WLP (V0)	74.25 ± 9.22	75.31 ± 6.30	0.706
	End of WLP (V6)	71.18 ± 10.17	71.96 ± 7.77	0.810
	End of P-WLP (V9)	72.07 ± 10.41	72.65 ± 6.82	0.855
	Dif V0–V6	−2.96 ± 1.79	−3.66 ± 2.60	0.732
	Dif V6–V9	0.79 ± 1.83	0.92 ± 2.59	0.633
	Rebound effect Dif V0–V9	−2.17 ± 2.59	−2.75 ± 3.27	0.806
Weight (kg) **OB1**	Baseline of WLP (V0)	84.77 ± 6.18	84.98 ± 8.77	0.942
	End of WLP (V6)	81.69 ± 5.56	79.71 ± 9.53	0.503
	End of P-WLP (V9)	83.33 ± 6.66	80.79 ± 10.32	0.442
	Dif V0–V6	−3.08 ± 1.73	−5.27 ± 2.76	0.017
	Dif V6–V9	1.64 ± 1.65	1.09 ± 3.45	0.594
	Rebound effect Dif V0–V9	−1.44 ± 2.51	−4.19 ± 3.61	0.026
BMI (kg/m^2^) **Overweight**	Baseline of WLP (V0)	28.12 ± 1.14	28.34 ± 1.02	0.567
	End of WLP (V6)	26.92 ± 1.27	27.05 ± 1.63	0.790
	9 months P-WLP (V9)	27.26 ± 1.61	27.34 ± 1.48	0.881
	Dif V0–V6	0.30 ± 0.72	0.37 ± 1.03	0.860
	Dif V6–V9	−1.16 ± 0.75	−1.41 ± 1.0	0.807
	Rebound effect Dif V0–V9	−0.85 ± 1.05	−1.01 ± 1.7	0.698
BMI (kg/m^2^) **OB1**	Baseline of WLP (V0)	31.98 ± 1.33	31.91 ± 1.22	0.883
	End of WLP (V6)	30.89 ± 1.65	29.91 ± 1.78**#**	0.136
	End of P-WLP (V9)	31.45 ± 1.99	30.30 ± 1.87	0.120
	Dif V0–V6	−1.09 ± 0.56	−1.99 ± 1.09	0.010
	Dif V6–V9	0.56 ± 0.63	0.4 ± 1.3	0.673
	Rebound effect Dif V0–V9	−0.52 ± 0.96	−1.61 ± 1.43	0.025
Fat mass (kg) **Overweight**	Baseline of WLP (V0)	26.55 ± 4.58	27.62 ± 3.69	0.481
	End of WLP (V6)	24.96 ± 3.53	26.19 ± 4.42	0.391
	End of P-WLP (V9)	25.37 ± 4.55	26.94 ± 5.10	0.364
	Dif V0–V6	−1.58 ± 3.75	−1.56 ± 2.76	0.982
	Dif V6–V9	0.40 ± 1,94	0.75 ± 2.72	0.685
	Rebound effect Dif V0–V9	−1.18 ± 3.66	−0.65 ± 3.63	0.804
Fat mass (kg) **OB1**	Baseline of WLP (V0)	32.91 ± 3.06	30.13 ± 7.78	0.222
	End of WLP (V6)	31.48 ± 3.43	28.48 ± 5.56	0.145
	End of P-WLP (V9)	33.54 ± 3.69	31.31 ± 5.57	0.217
	Dif V0–V6	−1.44 ± 3.29	−3.44±2.46	0.080
	Dif V6–V9	2.06 ± 1.62	2.02 ± 2.53	0.958
	Rebound effect Dif V0–V9	0.62 ± 3.80	−1.62 ± 3.45	0.121

Data are expressed as the means ± SDs. V0–V6: Difference during the WLP; V6–V9: Differences during the P-WLP; V0–V9: Rebound effect. WLP: Weight Loss Program; P-WLP: Post-weight Loss Program; BMI: Body Mass Index; OB1: Obesity type 1. #Intragroup difference V0 vs. V6 (*p* = 0.032).
